# Regression in hepatic fibrosis in elderly Chinese patients with hepatitis C receiving direct-acting antiviral treatment

**DOI:** 10.1186/s12876-023-02732-4

**Published:** 2023-04-03

**Authors:** Bin Niu, Wenqian Zang, Hui Zhou, Yuqiang Mi, Chengzhen Lu, Ping Li

**Affiliations:** 1 Clinical School of the Second People’s Hospital, Tianjin Medical University, Tianjin, 300192 China; 2 Department of Hepatology, Tianjin Second People’s Hospital, Tianjin, 300192 China; 3 Tianjin Research Institute of Liver Diseases, Tianjin, 300192 China

**Keywords:** Chronic hepatitis C (CHC), Direct-acting antiviral agents (DAAs), Liver fibrosis, Non-invasive evaluation, Liver stiffness measurement (LSM)

## Abstract

**Background:**

Patients infected with Hepatitis C virus (HCV) are recommended to receive treatment with direct-acting antiviral agents (DAAs), which have been certified to obtain a high sustained virological response (SVR). However, little is known about the benefits of successful anti-viral treatment to elderly patients with hepatic fibrosis. In this study, we aimed to assess degree of fibrosis in elderly patients with chronic hepatitis C (CHC) treated with DAAs, and to evaluate the correlations between identified factors associated with these changes.

**Methods:**

This study retrospectively enrolled elderly patients with CHC who received DAAs in Tianjin Second People’s Hospital from April 2018 to April 2021. The degree of liver fibrosis was assessed using serum biomarkers and transient elastography (TE) expressed as the liver stiffness (LSM), while the hepatic steatosis was evaluated by controlled attenuated parameter (CAP). Changes in factors related to hepatic fibrosis were examined following treatment with DAAs, and associated prognostic factors were further evaluated.

**Results:**

We included 347 CHC patients in our analysis, where 127 of these were elderly patients. For the elderly group, the median LSM was 11.6 (7.9–19.9) kPa, and this value was significantly reduced to 9.7 (6.2–16.6) kPa following DAA treatment. Similarly, GPR, FIB-4 and APRI indices were significantly reduced from 0.445 (0.275–1.022), 3.072 (2.047–5.129) and 0.833 (0.430–1.540) to 0.231 (0.155–0.412), 2.100 (1.540–3.034) and 0.336 (0.235–0.528), respectively. While in younger patients, the median LSM reduced from 8.8 (6.1–16.8) kPa to 7.2 (5.3–12.4) kPa, and the trends of GPR, FIB-4 and APRI were also consistent. The CAP in younger patients increased with statistical significance, but we did not observe any significant change in CAP for the elderly group. Based on multivariate analysis, age, LSM, and CAP before baseline were identified as determinants for LSM improvement in the elderly.

**Conclusion:**

In this study, we found that elderly CHC patients treated with DAA had significantly lower LSM, GPR, FIB-4, and APRI values. DAA treatment did not significantly change CAP. Furthermore, we observed correlations between three noninvasive serological evaluation markers and LSM. Finally, age, LSM, and CAP were identified as independent predictors of fibrosis regression in elderly patients with CHC.

## Background

Chronic hepatitis C (CHC) is a prevalent disease resulting from infection with hepatitis C virus (HCV), a hepatotropic RNA that can cause fibrosis, liver cirrhosis, and even hepatocellular carcinoma [1]. Approximately 71 million people across the world are infected with HCV, where the global prevalence of CHC is estimated to be 1.0%, and the reported incidences in China were 0.43% [2, 3]. Currently, the rate of increase in CHC is a relatively higher among elderly people, who typically have a longer course of disease, suffer from a higher amount of comorbidities and are more prone to adverse reactions. These factors significantly impact economic costs, morbidity and mortality on a global scale [4, 5].

Prior to 2011, the standard treatment for CHC was pegylated interferon (PEG-IFN) therapy [6]. However, PEG-IFN based therapy consistently leads to treatment intolerance, especially among elderly patients. Ever since the Food and Drug Administration (FDA) approved the first direct-acting antiviral agents (DAAs), new regiments involving DAAs have revolutionized the treatment of CHC. In fact, various forms of DAAs therapy have been recommended for all patients with chronic HCV infection [7–10]. DAAs were introduced in 2017 in most parts of mainland China, but the application has been limited due to the economic ability of patients in different regions, the relevant research data is lacking as well. However, thanks to the capitated payment policy in Tianjin, each HCV patient with Tianjin medical insurance can receive medical reimbursement for the total treatment cost. Not only is this financially convenient for patients, but it also provides good conditions for carrying out HCV-related research.

Currently, the degree of liver fibrosis is considered useful for predicting the development of liver fibrosis,cirrhosis, hepatocarcinoma even death in CHC patients following treatment, where its extent serves as an indicator of disease progression [11–13].

Although liver biopsy remains the gold standard for the evaluation and management of patients with fibrosis, its clinical application is largely limited in due to its invasiveness, inter-observer variability at pathologic evaluation, inadequate sampling size and sampling variations [14, 15].Instead, many non-invasive fibrosis indices have been applied to fibrosis assessment, these methods generally involve a physical approach based on the liver stiffness measurement (LSM) or a biological approach based on serum biomarkers [16, 17]. Of these techniques, transient elastography (TE) has gradually become the mainstay for non-invasive liver fibrosis evaluation, TE can be performed with excellent diagnostic accuracy and independent of the underlying liver disease for cirrhosis diagnosis [18]. Non-invasive scoring systems based on laboratory blood tests include the γ-GT-to-PLT ratio (GPR), AST-to-platelet ratio index (APRI) and fibrosis-4 (FIB-4) score. FIB-4 and APRI have demonstrated reliability in predicting cirrhosis during mass HCV treatment [19]. Although GPR scoring is a relatively new noninvasive evaluation method, this system has proven to be no less accurate than FIB-4 or APRI in staging liver fibrosis among patients with chronic hepatitis B [20].

However, the impact of DAAs on the elderly population has not garnered much attention. Existing research on DAAs rarely uses the elderly population as the main research object, rendering knowledge on DAA treatment among the elderly very limited.

Currently the available DAA regimens are well-tolerated and achieve high rates of sustained virological response (SVR) among elderly Chinese adults [21]. The purpose of this study was to evaluate liver fibrosis improvement resulting from DAA treatment in elderly patients with chronic HCV infection using various non-invasive measurements, and to further investigate the factors associated with these changes.

## Methods

### Study population and design

This retrospective cohort study enrolled all consecutive patients with chronic HCV infection treated with IFN-free DAA regimens at Tianjin Second People’s Hospital from April 2018 to April 2021. Treatment drugs and treatment course were determined according to the patient's genotype and hepatic function, with reference to the Guidelines for the prevention and treatment of hepatitis C (updated 2015 version and 2019 version) [5, 22].

The inclusion criteria used in this study were as follows: (1) age 18 years and above; (2) demonstrated presence of serum anti-HCV antibody for more than six months and detectable HCV RNA; (3) 12 weeks after completed the DAA treatment; (4) had measurement for GPR, FIB-4 and APRI, and underwent TE before and after treatment; and (5) Were treatment naïve or experienced.

The exclusion criteria were as follows: (1) showed presence of liver disease caused by other etiologies (, e.g., alcoholic liver disease, autoimmune hepatitis, drug-induced liver disease, Wilson disease, hemochromatosis, or a primary related diseases such as biliary cholangitis or primary sclerosing cholangitis); (2) concurrently used immunomodulatory agents, steroid hormones, or chemotherapy drugs; (3) previous drinking history or drinking alcohol during treatment; (4) used intravenous drugs during treatment; (5) were treated with a combination regimen including PEG-IFN; (6) were pregnancy and lactating.

Demographic and biochemical data, complete blood count analysis results, and assessments of LSM and controlled attenuated parameter (CAP) were collected before baseline and 12 weeks following the end of the DAAs treatment (EOT).

This study was conducted in accordance with the 1975 Declaration of Helsinki and approved by the Research Ethics Committee of Tianjin Second People’s Hospital (Jin Er Ren Min Lun Shen Zi [2021] No. 17).

### Patients grouping

Sixty year-old was chosen as the definition to differentiate the elderly patients and younger patients based on the World Health Organization’s reports, and the Elderly Rights Guarantees Law in China [23, 24]. Elderly patients were further divided into 2 subgroups according to whether they were 70 years or older.

Liver cirrhosis diagnosis was based on previous abdominal color Doppler ultrasound (Philips IU22-22,100, USA) or liver biopsy.

### Sustained virological response measurement (SVR)

SVR was defined as an HCV RNA level below the limit of quantitation or undetected 12 weeks after the end of antiviral therapy. HCV RNA was measured using the COBAS AmpliPrep/COBAS TaqMan48 (lower limit of detection, 15 IU/ml) (Roche, Switzerland).

### Patients’ data collection and laboratory tests

We collected demographic characteristics and clinical data from the included study subjects, such as age, gender, DAAs regiment, history of prior treatment and concomitant comorbidities.

Venous blood was taken from fasted patients to test its laboratory indicators: Alanine aminotransferase (ALT), aspartate aminotransferase (AST), albumin (ALB), gamma-glutamyl transferase (γ-GT) and total bilirubin (TBIL) were detected using the Japanese HITACH1 automatic biochemical analyzer-7180 (reagent from HeGuang Kabuskiki Kaisha, Japan); White blood cell (WBC) and, platelet (PLT) counts were measured using the Japanese automatic blood cell analyzer SysmexXN-2000 (reagents purchased from Sysmex Europe GmbH, Germany). The above clinical examination operations were carried out by professional technicians according to the operating instructions.

### Liver fibrosis indices

This study used GPR, FIB-4, APRI and TE to assess the degree of liver fibrosis. The liver fibrosis indices were calculated as follows:

- GPR = (γ-GT [IU/L]/upper limit of normal γ-GT [IU/L]) *100/platelet count (10^9^/L) [20].
- FIB-4 = AST (IU/L) * age (years)/platelet count (10^9^/L) * ALT (IU/L) [25].
- APRI = (AST [IU/L]/upper limit of normal AST [IU/L]) *100/platelet count (10^9^/L) [26].

Liver stiffness was evaluated through one-dimensional ultrasound TE (FibroScan-502, Echosens, French), LSM is expressed in kilopascals (kPa), and CAP is expressed in decibels per meter (dB/m).

TE was performed on the patient lying supine with their right arm elevated and breath held, in order to facilitate access to the right liver lobe away while avoiding large vessels. Participants in this study were considered to have completed a valid TE if the following criteria were fulfilled: 1) number of valid shots of at least 10; 2) success rate (ratio of valid shots to total shots) above 60%; and 3) interquartile range (IQR, reflecting the variability of measurements) less than 30% of the median LSM value (IQR/M ≤ 0.30%) [17].

### Statistical analysis

Count data is expressed as frequency or ratio (%). Categorical variables were assessed using the Chi-square or Fisher’s exact test. Measurement data were tested for normality prior to statistical testing. Normal variables were expressed as‾X ± SD, while skewed variables are expressed as median (25^th^ percentile-75^th^ percentile). The *t*-test, Mann–Whitney *U* test or Wilcoxon signed-rank test was used to analyze differences between groups. Factors associated with changes in fibrosis were accessed using univariate and logistic regression analysis. All statistical analyses were performed using SPSS 22.0 and GraphPad Prism 6 statistical software. *P* < 0.05 was considered statistically significant.

## Results

### Patient enrollment

**Fig. 1:**
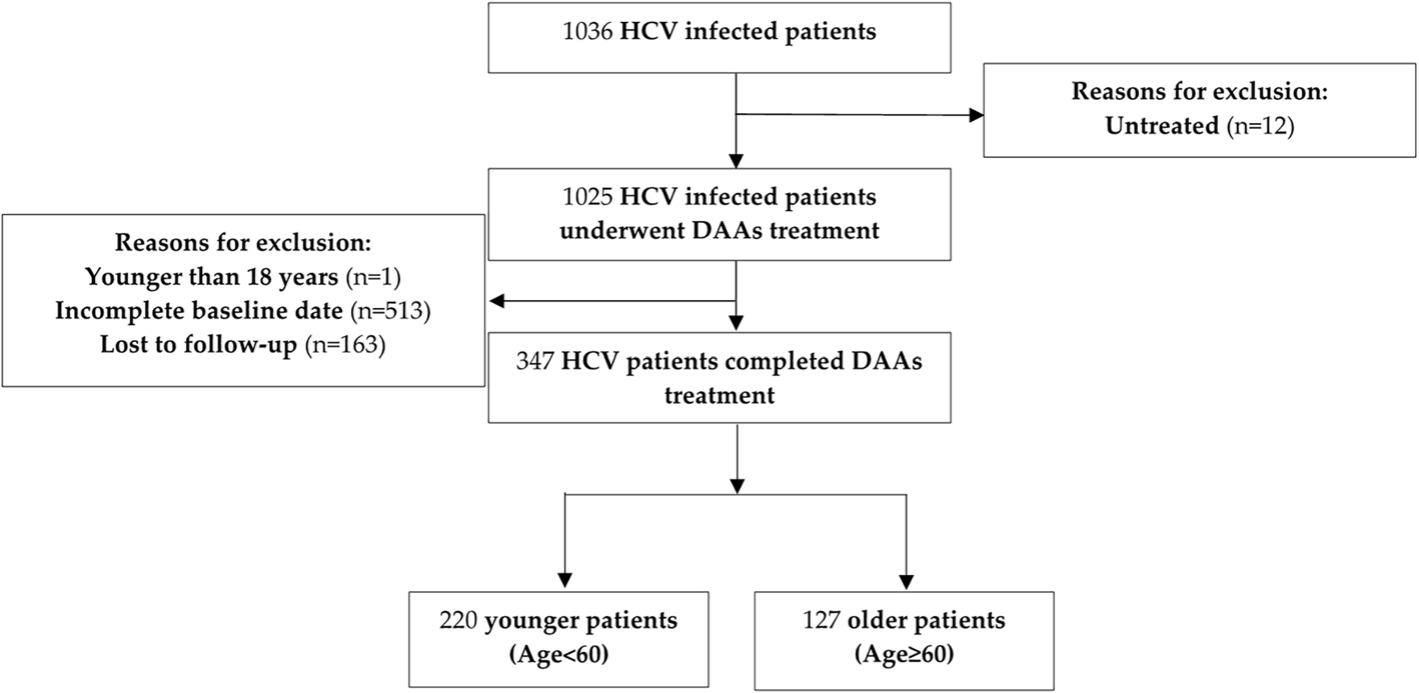
Flowchart of patient enrollment

A total of 1036 patients with chronic HCV infection met the initial screening criteria for this study. We excluded patients who were younger than 18 (*n* = 1), had inadequate baseline date (*n* = 513), were untreated (*n* = 12), or were, lost to follow-up (*n* = 163). Finally, a total of 347 patients receiving DAA treatment for CHC were included, of which 127 patients were defined as elderly individuals. Figure 1 depicts the the flowchart of patient enrollment for this study.

### Patients’ characteristics

Of the 347 enrolled patients, 220 were considered younger and 127 were elderly. We did not observe any differences between the two groups in terms of demographic characteristics, such as gender or initial treatment. Additionally, DAA regimen, and HCV RNA load at each age did not significantly differ between the groups. However, genotype 3 and 6 was more common in younger patients compared to elderly patients. The SVR for younger patients was seemingly better than that of the elderly, but this difference was not significant. We observed significantly lower ALB, WBC, and PLT levels in the elderly group. Elderly patients were also more prone to comorbidities, such as hypertension and diabetes. The elderly group had significantly higher LSM, FIB-4 and APRI compared to younger patients, while the two groups did not significantly differ in terms of CAP or GPR. Table 1 compares patients characteristics between the two groups.

**Table 1:** Demographic and clinical characteristics of the patients

Characteristics	Total (*n* = 347)	Younger patients (*n* = 220)	Elderly patients (*n* = 127)	*P* value
Age	56 (47–63)	47 (24–59)	65 (62–70)	**< 0.001**
Gender, count (male, %)	147 (42.4%)	101 (45.9%)	46 (36.2%)	0.079
Cirrhosis, count (%)	213 (61.4%)	132 (60.0%)	81 (63.8%)	0.486
Treatment-experienced, count No (%)	9 (2.6%)	3 (1.4%)	6 (4.7%)	0.122
History of HCC, count (%)	3 (0.9%)	0 (0.0%)	3 (2.4%)	**0.022**
Genotype, count (%)				**0.003**
1	249 (71.8%)	149 (67.7%)	100 (78.7%)	**0.028**
2	67 (19.3%)	42 (19.1%)	25 (19.7%)	0.893
3	22 (6.3%)	20 (9.1%)	2 (1.6%)	**0.006**
6	8 (2.3%)	8 (3.6%)	0 (0.0%)	**0.029**
Mixed/ indeterminate	1 (0.3%)	1 (0.5%)	0 (0.0%)	1.000
High HCV-RNA load, count No (%)	271 (78.1%)	171 (77.7%)	100 (78.7%)	0.826
SVR, No (%)	342 (98.6%)	219 (99.5%)	123 (96.9%)	0.062
ALT (U/L)	42.0 (22.0–69.0)	43.0 (22.3–68.8)	37.0 (21.0–69.0)	0.351
AST (U/L)	38.0 (25.0–59.8)	37.0 (24.0–57.0)	41.0 (26.0–64.0)	0.162
ALB (g/L)	44.2 (41.0–46.7)	44.8 (41.7–48.3)	43.3 (40.0–45.9)	** < 0.001**
γ-GT (U/L)	41.0 (25.0–82.0)	42.0 (27.0–92.0)	40.0 (24.0–62.0)	0.148
TBil (μmol/L)	14.7 (11.1–19.0)	14.1 (10.6–18.1)	15.8 (12.9–20.5)	**0.006**
WBC (10^9^/L)	4.86 (4.00–6.25)	5.11 (4.04–6.63)	4.61 (3.88–5.60)	**0.007**
PLT (10^9^/L)	162 (112–218)	180 (193–254)	141 (104–190)	** < 0.001**
LSM (kPa)	10.0 (6.8–17.6)	8.8 (6.1–16.8)	11.6 (7.9–19.9)	** < 0.001**
CAP (dB/m)	225(195–256)	225(193–255)	230(200–258)	0.213
GPR	0.464 (0.247–1.018)	0.475 (0.220–1.015)	0.445 (0.275–1.022)	0.743
FIB-4	2.047 (1.278–3.795)	1.565 (0.976–2.818)	3.072 (2.047–5.129)	** < 0.001**
APRI	0.661 (0.373–1.250)	0.552 (0.343–1.123)	0.833 (0.430–1.540)	**0.003**
Treatment regimen, count (%)				0.470
SOF ± RBV	190 (54.8%)	122 (55.5%)	68 (53.5%)	
SOF + DCV ± RBV	11 (3.2%)	5 (2.3%)	6 (4.7%)	
OBV/PTV/r/DSV ± RBV	50 (14.4%)	30 (13.6%)	20 (15.7%)	
DCV + ASV	6 (1.7%)	3 (1.4%)	3 (2.2%)	
DNVr + DCV ± RBV	20 (5.8%)	16 (7.3%)	4 (3.1%)	
LEV/SOF	19 (5.5%)	10 (4.5%)	9 (7.1%)	
EBR/GZR	28 (8.1%)	20 (9.1%)	8 (6.3%)	
SOF/VEL	23 (6.6%)	14 (6.4%)	9 (7.1%)	
Comorbidity, count (%)				
Hypertension	61 (17.6%)	29 (13.2%)	32 (25.4%)	**0.004**
Diabetes	37 (10.7%)	15 (6.8%)	22 (17.5%)	**0.002**
Cardiovascular disease	15 (4.3%)	7 (3.2%)	8 (6.3%)	0.169
Chronic kidney disease	5 (1.4%)	3 (1.4%)	2 (1.6%)	1.000
HBV coinfection	5 (1.4%)	3 (1.4%)	2 (1.6%)	1.000
HIV coinfection	3 (0.9%)	3 (1.4%)	0 (0.0%)	0.302

*HCC* Hepatocellular carcinoma, *HCV* Hepatitis C virus, *SVR* Sustained virologic response, *ALT* Alanine aminotransferase, *AST* Aspartate aminotransferase, *ALB* Serum albumin, *γ-GT* Gamma-glutamyl transferase, *TBil* Total bilirubin, *WBC* White blood cell, *PLT* Platelet, *LSM* Liver stiffness measurement, *CAP* Controlled attenuation parameter, *GPR* γ-glutamyl transpeptidase-to- platelet ratio, *FIB-4* Fibrosis-4, *APRI* Aspartate aminotransferase-to-platelet ratio index, *SOF* Sofosbuvir, *RBV* Ribavirin, *DCV* Daclatasvir, *OBV/PTV/r/DSV* Ombitasvir/paritaprevir/ritonavir/dasabuvir, *ASV* Asunaprevir, *DNVr* Danoprevir/ ritonavir, *LEV/SOF* Ledipasvir/sofosbuvir, *EBR/GZR* Elbasvir/grazoprevir, *SOF/VEL* Sofosbuvir/velpatasvir, *HBV* Hepatitis B virus, *HIV* Human immunodeficiency virus

We further divided the elderly patients into following two subgroups: (I) the 60–69 age group; and (II) the ≥ 70 age group. Table 2 shows that the majority of demographic characteristics and laboratory parameters for these two subgroups did not significantly differ. In patients aged ≥ 70, LSM, CAP, GPR and FIB-4 were higher for patients aged 60 to 69, whereas APRI was lower for this group-however, none of these differences were significant.

**Table 2:** Demographic and clinical characteristics of the elderly patients (age ≥ 60 years)

Characteristics	Elderly patients (*n* = 127)	Age 60–69 (*n* = 97)	Age ≥ 70 (*n* = 30)	*P* value
Age	65 (62–70)	63 (62–66)	74 (71–75)	**< 0.001**
Gender, count (male, %)	46 (36.2%)	33 (34.0%)	13 (28.3%)	0.354
Cirrhosis, count (%)	81 (63.8%)	62 (63.9%)	19 (63.3%)	0.954
Treatment-experienced, count No (%)	6 (4.7%)	3 (3.1%)	3 (10.0%)	0.143
History of HCC, count (%)	3 (2.4%)	3 (3.1%)	0 (0.0%)	1.000
Genotype, count (%)				0.554
1	100 (78.7%)	78 (80.4%)	22 (73.3%)	
2	25 (19.7%)	18 (18.6%)	7 (23.3%)	
3	2 (1.6%)	1 (1.0%)	1 (3.3%)	
6	0 (0.0%)	0 (0.0%)	0 (0.0%)	
Mixed/ indeterminate	0 (0.0%)	0 (0.0%)	0 (0.0%)	
High HCV-RNA load, count No (%)	100 (78.7%)	74 (76.3%)	26 (86.7%)	0.225
SVR, No (%)	123 (96.9%)	94 (96.9%)	29 (96.7%)	1.000
ALT (U/L)	37.0 (21.0–69.0)	38.0 (23.0–69.0)	35.5 (18.8–76.0)	0.462
AST (U/L)	41.0 (26.0–64.0)	41.0 (27.5–69.4)	39.0 (21.0–58.5)	0.296
ALB (g/L)	43.3 (40.0–45.9)	43.6 (39.9–46.2)	42.8 (40.0–45.3)	0.445
γ-GT (U/L)	40.0 (24.0–62.0)	37.0 (23.5–59.0)	44.5 (23.3–107.0)	0.559
TBil (μmol/L)	15.8 (12.9–20.5)	15.8 (12.9–20.3)	15.3 (11.7–21.8)	0.918
WBC (10^9^/L)	4.61 (3.88–5.60)	4.61 (3.67–5.63)	4.63 (4.20–5.63)	0.576
PLT (10^9^/L)	141 (104–190)	138 (104–194)	162 (102–185)	0.845
LSM (kPa)	11.6 (7.9–19.9)	11.2 (7.7–19.6)	14.1 (9.8–23.2)	0.184
CAP (dB/m)	230(200–258)	223(197–257)	239(210–281)	0.099
GPR	0.445 (0.275–1.022)	0.430 (0.274–1.018)	0.512 (0.313–1.026)	0.540
FIB-4	3.072 (2.047–5.129)	3.041 (1.973–5.020)	3.572 (2.163–6.096)	0.536
APRI	0.833 (0.430–1.540)	0.841 (0.465–1.604)	0.785 (0.371–1.355)	0.417
Treatment regimen, count (%)				0.319
SOF ± RBV	68 (53.5%)	57 (58.8%)	11 (36.7%)	
SOF + DCV ± RBV	6 (4.7%)	4 (4.1%)	2 (6.7%)	
OBV/PTV/r/DSV ± RBV	20 (15.7%)	15 (15.5%)	5 (16.7%)	
DCV + ASV	3 (2.2%)	1 (1.0%)	2 (6.7%)	
DNVr + DCV ± RBV	4 (3.1%)	3 (3.1%)	1 (3.3%)	
LEV/SOF	9 (7.1%)	5 (5.2%)	4 (13.3%)	
EBR/GZR	8 (6.3%)	6 (6.2%)	2 (6.7%)	
SOF/VEL	9 (7.1%)	6 (6.2%)	3 (10.0%)	
Comorbidity, count (%)				
Hypertension	32 (25.4%)	24 (24.7%)	8 (27.6%)	0.758
Diabetes	22 (17.5%)	12 (12.4%)	10 (34.5%)	**0.006**
Cardiovascular disease	8 (6.3%)	6 (6.2%)	2 (6.7%)	1.000
Chronic kidney disease	2 (1.6%)	1 (1.0%)	1 (3.3%)	0.418
HBV coinfection	2 (1.6%)	1 (1.0%)	1 (3.3%)	0.418
HIV coinfection	0 (0.0%)	0 (0.0%)	0 (0.0%)	-

*HCC* Hepatocellular carcinoma, *HCV* Hepatitis C virus, *SVR* Sustained virologic response, *ALT* Alanine aminotransferase, *AST* Aspartate aminotransferase, *ALB* Serum albumin, *γ-GT* Gamma-glutamyl transferase, *TBil* Total bilirubin, *WBC* White blood cell, *PLT* Platelet, *LSM* Liver stiffness measurement, *CAP* Controlled attenuation parameter, *GPR* γ-glutamyl transpeptidase-to- platelet ratio, *FIB-4* Fibrosis-4, *APRI* Aspartate aminotransferase-to-platelet ratio index, *SOF* Sofosbuvir, *RBV* Ribavirin, *DCV* Daclatasvir, *OBV/PTV/r/DSV* Ombitasvir/paritaprevir/ritonavir/dasabuvir, *ASV* Asunaprevir, *DNVr* Danoprevir/ ritonavir, *LEV/SOF* Ledipasvir/sofosbuvir, *EBR/GZR* Elbasvir/grazoprevir, *SOF/VEL* Ssofosbuvir/velpatasvir, *HBV* Hepatitis B virus, *HIV* Human immunodeficiency virus

### Fibrosis measurements

Table 3 summarizes the liver fibrosis measurements for the patients before and after DAA treatment. As previously mentioned, the LSM, FIB-4, and APRI values before DAA treatment were significantly higher in the elderly groups compared to the younger groups, while the CAP and GPR values did not significantly differ. The initial liver fibrosis measurements did not significantly differ between the two elderly subgroups, FIB-4 and APRI remained significantly higher in the elderly group following DAA treatment.

**Table 3:** Changes in noninvasive measurements of liver fibrosis in the patients

Variable	Total (*n* = 347)	Total			Elderly patients		
		Younger patients (*n* = 220)	Elderly patients (*n* = 127)	*P* value^b^	Age 60–69 (*n* = 97)	Age ≥ 70 (*n* = 30)	*P* value^c^
LSM (kPa)							
Pre-DAAs	10.0 (6.8–17.6)	8.8 (6.1–16.8)	11.6 (7.9–19.9)	**< 0.001**	11.2 (7.7–19.6)	14.1 (9.8–23.2)	0.184
Post-DAAs	7.8 (5.5–13.8)	7.2 (5.3–12.4)	9.7 (6.2–16.6)	**< 0.001**	8.9 (6.1–15.4)	10.7 (8.0–21.0)	0.115
ΔLSM^a^	1.7 (-0.4–4.9)	1.6 (-0.5–4.4)	2.1 (-0.3–5.6)	0.287	2.1 (-0.2–5.4)	2.6 (-0.9–7.8)	0.907
CAP (dB/m)							
Pre-DAAs	225(195–256)	225(193–255)	230(200–258)	0.213	223(197–257)	239(210–281)	0.099
Post-DAAs	235(207–272)	236(208–273)	235(204–269)	0.824	232(201–264)	240(217–291)	0.194
Δ CAP^a^	-11 (-45–22)	-17 (-53–21)	-4 (-42–23)	0.183	-4 (-42–23)	-8 (-45–23)	0.964
GPR							
Pre-DAAs	0.464 (0.247–1.018)	0.475 (0.220–1.015)	0.445 (0.275–1.022)	0.743	0.430 (0.274–1.018)	0.512 (0.313–1.026)	0.540
Post-DAAs	0.214 (0.133–0.384)	0.203 (0.125–0.382)	0.231 (0.155–0.412)	0.164	0.215 (0.153–0.416)	0.267 (0.170–0.338)	0.473
Δ GPR^a^	0.197 (0.051–0.532)	0.198(0.055–0.538)	0.195 (0.046–0.519)	0.924	0.199 (0.050–0.515)	0.180 (0.042–0.608)	0.851
FIB-4							
Pre-DAAs	2.047 (1.278–3.795)	1.565 (0.976–2.818)	3.072 (2.047–5.129)	** < 0.001**	3.041 (1.973–5.020)	3.572 (2.163–6.096)	0.536
Post-DAAs	1.522 (0.942–2.595)	1.139 (0.788–1.878)	2.100 (1.540–3.034)	** < 0.001**	2.012 (1.473–2.865)	2.370 (1.881–3.517)	0.066
Δ FIB-4^a^	0.366 (0.018–1.075)	0.271 (0.003–0.715)	0.637 (0.137–1.647)	** < 0.001**	0.626 (0.166–1.827)	0.762 (-0.474–1.733)	0.807
APRI							
Pre-DAAs	0.661 (0.373–1.250)	0.552 (0.343–1.123)	0.833 (0.430–1.540)	**0.003**	0.841 (0.465–1.604)	0.785 (0.371–1.355)	0.417
Post-DAAs	0.283 (0.194–0.472)	0.241 (0.178–0.450)	0.336 (0.235–0.528)	** < 0.001**	0.324 (0.218–0.528)	0.348 (0.242–0.508)	0.600
Δ APRI^a^	0.282 (0.087–0.688)	0.261 (0.077–0.620)	0.367 (0.110–0.900)	**0.046**	0.427 (0.123–0.895)	0.297 (0.074–0.943)	0.496

*DAAs* Direct-acting antiviral agents, *LSM* Liver stiffness measurement, *CAP* Controlled attenuation parameter, *GPR* γ-glutamyl transpeptidase-to- platelet ratio; FIB-4, Fibrosis-4; APRI, aspartate aminotransferase-to-platelet ratio index

^a^Δ value = Pre-DAAs value—Post-DAAs value

^b^*P* value = Younger patients versus Elderly patients

^c^*P* value = Age 60–69 versus Age ≥ 70

The absolute changes in liver fibrosis indices are also shown in Table 3. All indices besides CAP decreased after DAA treatment. Changes for FIB-4 and APRI were more dramatic in the elderly group of patients.

**Fig. 2:**
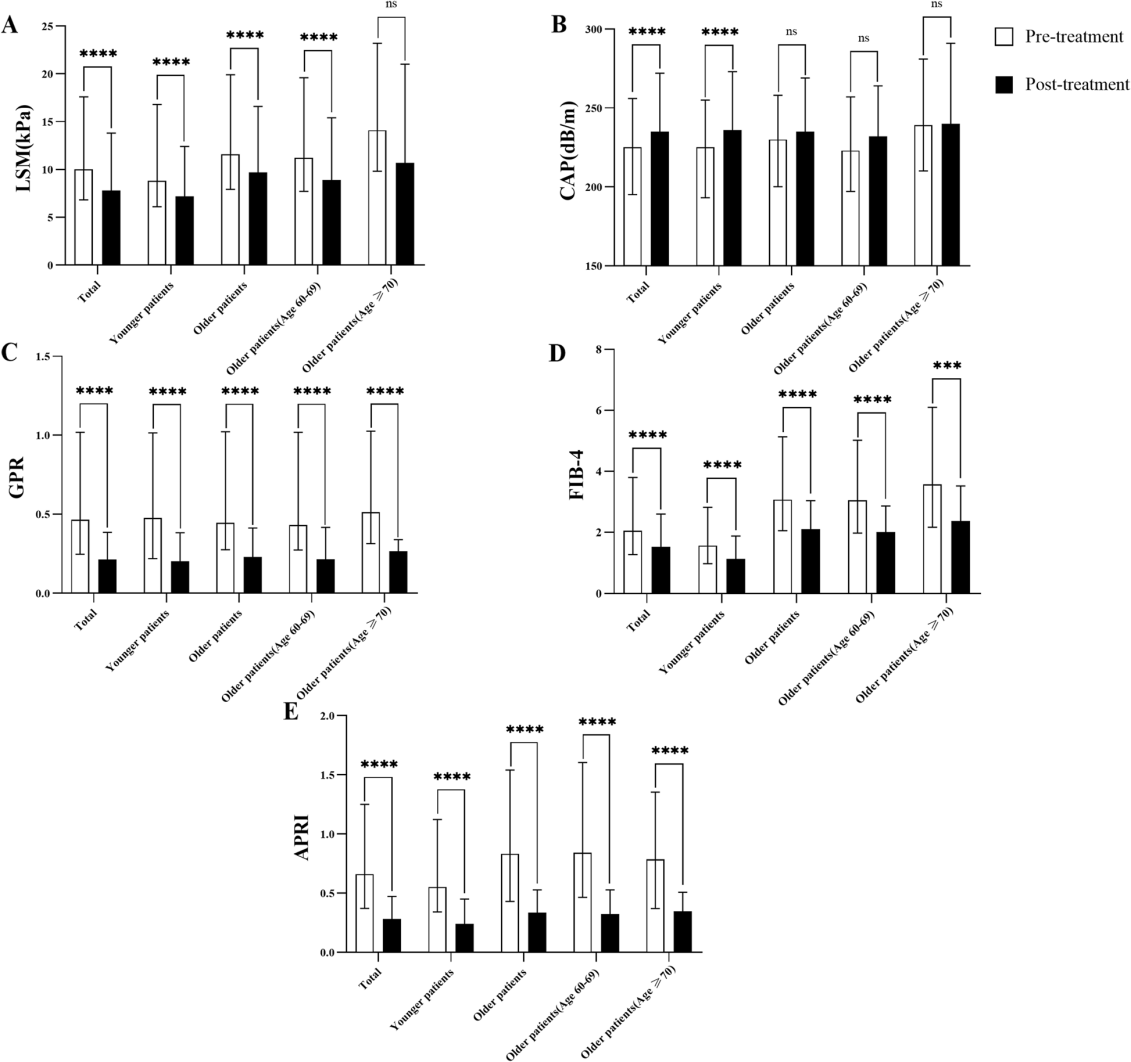
Changes in (**A**) LSM, (**B**) CAP, (**C**) GPR, (**D**) FIB-4, and (**E**) APRI following DAA treatment. ****P ≤ 0.0001; ***P ≤ 0.001; **P ≤ 0.01; *P ≤ 0.05; ^ns^*P* ≥ 0.05

Figure 2 demonstrates that the LSM, GPR, FIB-4, and APRI values decreased following treatment in all groups. CAP followed the opposite trend.

For the entire group of patients, the baseline median LSM value decreased after treatment with DAAs (*P* < 0.001). We observed a significant decrease in LSM for elderly patients (*P* < 0.001). However, in patients over 70 years old, LSM did not significantly change after treatment (*P* = 0.084).

For all patients, CAP increased notably from a relative low baseline median value. In the younger group, the median value at baseline was 225 (193–255) dB/m, which increased remarkedly following treatment (*P* < 0.001). In the elderly group, CAP did not significantly differ before versus after treatment (*P* = 0.56).

Among all patients, GPR, FIB-4, and APRI illustrated reduction following DAA treatment with statistically significant, respectively. We also observed significant decreases in these values for the entire elderly group and subgroup of patients over the age of 70.

### Correlation between liver fibrosis indices and LSM

**Fig. 3:**
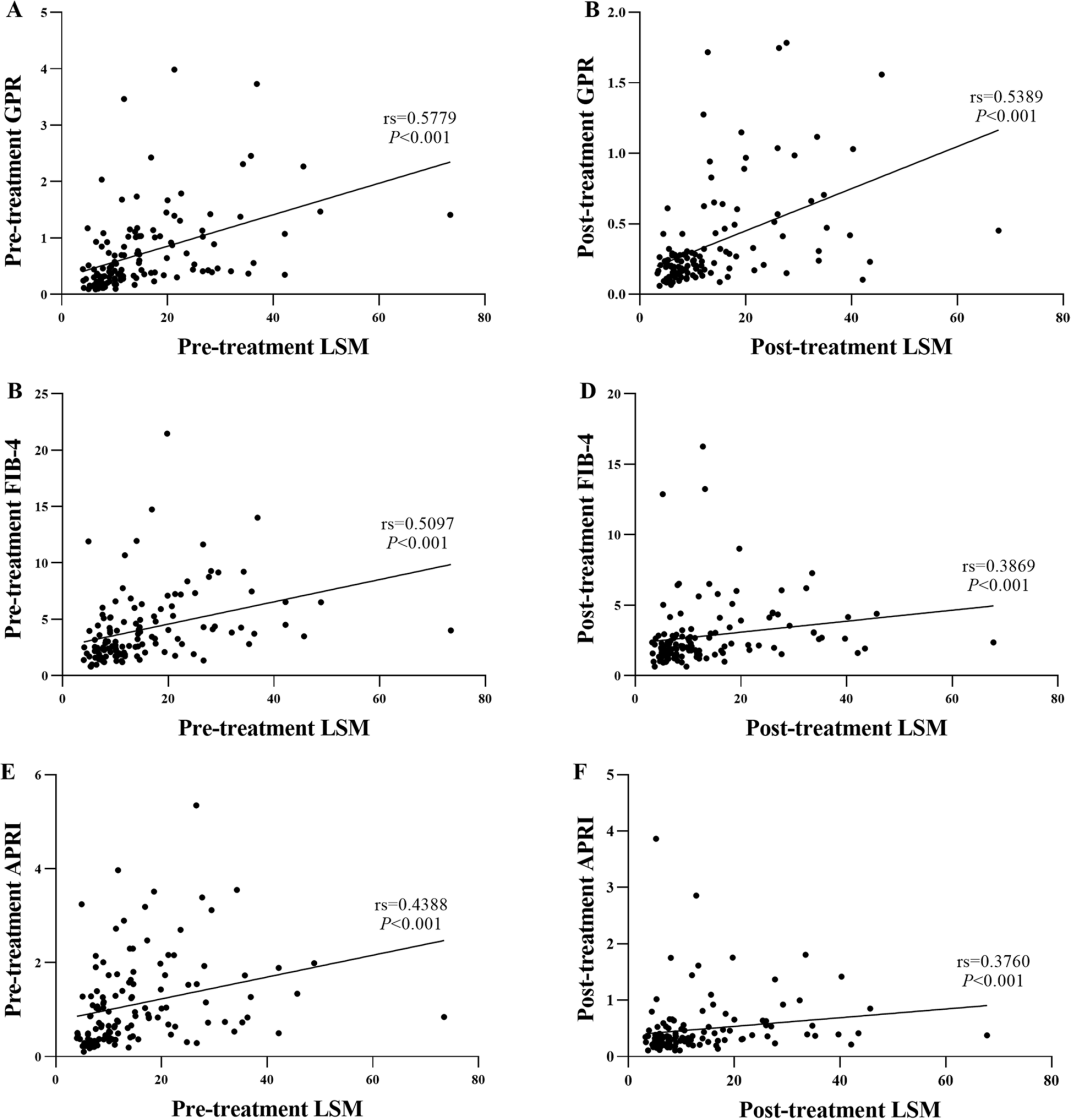
Correlations between (**A**) GPR and LSM before DAA treatment, (**B**) GPR and LSM after DAA treatment, (**C**) FIB-4 and LSM before DAA treatment, (**D**) FIB-4 and LSM after DAA treatment, (**E**) APRI and LSM before DAA treatment, and (**F**) APRI and LSM after DAA treatment

Figure 3 and Table 4 illustrated the relationships between the different liver fibrosis indices and LSM. GPR, FIB-4, and APRI were found to have individual correlations with LSM in the older group, both before and after treatment with DAAs.

**Table 4:** Correlations between the methods to check the fibrosis

Correlation	LSM	
	Pre-treatment	Post-treatment
GPR	0.5779*	0.5389*
FIB-4	0.5097*	0.3869*
APRI	0.4388*	0.3760*

*LSM* Liver stiffness measurement, *GPR* γ-glutamyl transpeptidase-to- platelet ratio, *FIB-4* Fibrosis-4, *APRI* Aspartate aminotransferase-to-platelet ratio index. *:*P* ≤ 0.001

We observed a significant correlation between GPR and LSM both before and after DAA treatment (*P* < 0.001). FIB-4 and LSM were also significantly correlated both before and after treatment (*P* < 0.001). Finally, APRI and LSM were significantly correlated both before and after treatment (*P* < 0.001).

### Associated factors with LSM

Table 5 summarizes the factors associated with fibrosis regression via univariate analysis. For the elderly group, LSM and CAP were significantly associated with successful fibrosis regression by univariate analysis. Variables that were considered clinically relevant or demonstrated a univariate relationship with LSM improvement were entered into the multivariate regression model. Given the number of events available, several variables were carefully chosen, including age, LSM, CAP, GPR, FIB-4, APRI, and diabetes.

**Table 5:** Factors associated with univariate regression of liver stiffness after the end of treatment in elderly patients

Variable	Univariate analysis	
	OR (95% CI)	*P* value
Age	0.937 (0.869–1.010)	0.089
Gender	0.629 (0.277–1.427)	0.267
Cirrhosis	1.339 (0.598–3.001)	0.478
Treatment-experienced	2.457 (0.473–12.767)	0.285
History of HCC	4.889 (0.430–55.620)	0.201
Genotype	n/a	0.264
High HCV-RNA load	1.018 (0.402–2.580)	0.970
SVR	2.417 (0.328–17.823)	0.387
ALT (U/L)	1.007 (0.995–1.018)	0.256
AST (U/L)	1.011 (0.997–1.025)	0.128
ALB (g/L)	1.004 (0.935–1.078)	0.910
γ-GT (U/L)	1.008 (0.997–1.019)	0.139
TBil (μmol/L)	1.005 (0.970–1.043)	0.769
WBC (10^9^/L)	1.081 (0.866–1.326)	0.493
PLT (10^9^/L)	1.000 (0.994–1.006)	0.993
LSM (kPa)	1.054 (1.004–1.107)	**0.033**
CAP (dB/m)	0.990 (0.982–0.999)	**0.021**
GPR	1.202 (0.674–2.143)	0.533
FIB-4	1.082 (0.941–1.244)	0.271
APRI	1.158 (0.751–1.785)	0.508
Treatment regimen	n/a	0.920
Hypertension	1.128 (0.472–2.695)	0.786
Diabetes	0.633 (0.215–1.862)	0.406
Cardiovascular disease	2.500 (0.591–10.571)	0.213

*HCC* Hepatocellular carcinoma, *HCV* Hepatitis C virus, *SVR* Sustained virologic response, *ALT* Alanine aminotransferase, *AST* Aspartate aminotransferase, *ALB* serum Albumin, *γ-GT* Gamma-glutamyl transferase, *TBil* Total bilirubin, *WBC* White blood cell, *PLT* Platelet, *LSM* Liver stiffness measurement, *CAP* Controlled attenuation parameter, *GPR* γ-glutamyl transpeptidase-to- platelet ratio, *FIB-4* Fibrosis-4, *APRI* Aspartate aminotransferase-to-platelet ratio index

As evident from our multivariate logistic regression analysis show in Table 6, a higher LSM value and lower CAP value remained significant after controlling for the other listed covariates. Additionally, age was found to be independently associated with LSM improvement.

**Table 6:** Factors associated with multivariate regression of liver stiffness after the end of treatment in elderly patients

Variable	Multivariate analysis	
	OR (95% CI)	*P* value
Age	0.916 (0.840–0.999)	**0.049**
LSM (kPa)	1.062 (1.003–1.125)	**0.038**
CAP (dB/m)	0.990 (0.981–0.999)	**0.029**
GPR	0.701 (0.311–1.581)	0.392
FIB-4	1.122 (0.836–1.506)	0.443
APRI	0.799 (0.342–1.867)	0.605
Diabetes	0.477 (0.142–1.600)	0.230

*LSM* Liver stiffness measurement, *CAP* Controlled attenuation parameter, *GPR* γ-glutamyl transpeptidase-to- platelet ratio, *FIB-4* Fibrosis-4, *APRI* Aspartate aminotransferase-to-platelet ratio index

## Discussion

In this study, we found that LSM, GPR, FIB-4, and APRI in CHC patients decreased after DAAs treatment whether the patient is elderly or not, which indicates regression of fibrosis. Besides, the CAP did not change significantly in the elderly group. Among the elderly, the correlations between three noninvasive serological evaluation markers and LSM were positive and significant. Meanwhile, this study also showed that age, LSM and CAP were independent predictors of fibrosis regression based on multivariate analysis.

To the best of our knowledge, this was the first study to explore the effect of DAA treatment on liver fibrosis in elderly Chinese patients with CHC. The main reasons for using DAAs to treat CHC include preventing the development of liver fibrosis and improving existing liver fibrosis status [5]. According to current knowledge, the regression of fibrosis is mainly achieved through the elimination of HCV. [27] Treatment with either IFN or DAAs has the capacity to achieve SVR, and many articles have proven that IFN-based treatment can significantly improve the degree of fibrosis [28–30]. Nevertheless, IFN-based therapy has been largely replaced by DAA treatment, which has caused the treatment of CHC to enter a pan-genotypic era. Our experience with and knowledge of DAA treatment is still rapidly evolving.

Since DAAs were first approved by FDA, multiple studies have demonstrated their role in improving the degree of liver fibrosis. Bachhofner et al. observed a significant reduction in liver stiffness after DAA-based treatment, which corresponded to a TE regression of more than 30% [31]. Similarly, Knop V et al. found that DAA treatment caused 88% of their patients to experience a reduction in liver stiffness [32]. Notably, most of the relevant studies were carried out in European and American countries. There is a lack of relevant data in China, and especially in the mainland region. However, some clinical trials conducted in China have confirmed the improvement in liver fibrosis resulting from DAAs. In their cohort of 102 patients with CHC, Kang Q et al. observed a decrease in median LSM from a baseline value of 10.45 kPa to 7.60 kPa following DAA treatment; significant decreases were also observed for FIB-4 and APRI scores [33]. In their study, Huang R et al. enrolled 40 patients treated with DAAs, finding that LSM, FIB-4, and APRI achieved statistically significant improvements by liver biopsy [34].

Currently, it has been proven that the available DAAs regimens are well-tolerated and could achieve high rates of sustained virological response (SVR) in Chinese elderly adults [21]. However, none of these studies paid special attention to elderly patients – in fact, the oldest participant was only 67 years old. Since elderly patients often suffer from a variety of basic diseases or take a variety of drugs at the same time, this group does not typically partake in clinical trials. Therefore, the current body of research may not be applicable to this specific sub-population. Our study aimed to fill the lack of data in this area.

Although liver biopsy is recommended prior to antiviral treatment, it may cause complications and is limited by relatively high costs, meaning it is not typically used in the routine management of CHC patients receiving DAAs. To replace biopsy, several noninvasive tests have been created, which are reproducible, and validated in several published studies [35, 36]. Accordingly, we applied TE to measure LSM, and GPR, FIB-4, and APRI scores to evaluate the degree of liver fibrosis.

Consistent with the results of the aforementioned reports, we found that the baseline level of LSM was higher in elderly patients compared to young patients, but no significant difference was observed between the subgroups of patients over 60 years old. Regardless of whether the patients were elderly, this study showed that DAA treatment improves LSM. However, although patients over 70 were found to achieve fibrosis regression, they did not demonstrate a statistically significant decrease in LSM. Among all patients, GPR, FIB-4, and APRI were significantly improved after DAA treatment. We also evaluated the correlations between serum biomarkers and LSM in elderly patients. GPR, FIB-4, and APRI were found to have correlations with TE measurement both before and after treatment, although these relationships were not particularly strong.

While the GPR, APRI, and FIB-4 values rapidly improved after DAA therapy from baseline to 12 weeks, these changes partly reflect improvement of necroinflammation rather than fibrosis regression completely because of the limitations of biochemical indicators. Similarly, TE measurement has been proven to be affected by necro-inflammatory activity to a certain extent, and especially during the onset of acute hepatitis [37]. However, the patients in this study had relatively stable liver disease, and most of their liver enzymes were less than three times the normal upper limit, meaning these factors can be considered to have little on LSM [38]. Furthermore, Lens S, et al. conducted a multicenter study, in which the hepatic venous pressure gradient (HVPG) of patients with HCV-associated cirrhosis significantly decreased after antiviral therapy [39]. Despite the lack of evidence of liver histology, this study reasonably speculated that DAAs did improve the degree of liver fibrosis and not simply inflammation and necrosis.

HCV infection can activate the sterol regulatory element-binding protein (SREBP) signaling pathway, resulting in a temporary increase in its expression level that further increases the adipogenesis of cholesterol and membrane lipids [40]. Theoretically, HCV eradication by DAA treatment is expected to regulate the expression of SREBP and reduce adipogenesis in the liver, which subsequently reduces CAP, an alternative marker of hepatic steatosis. Some previous studies have confirmed this conjecture [41, 42]. However, recent reports have also shown that successful HCV eradication by DAA therapy may cause an elevation in CAP [43, 44]. In this study, we assessed the degree of hepatic steatosis using CAP, finding that the CAP value was higher in elderly patients compared to young patients both before and after treatment. Additionally, the CAP value for patients over 70 years old was higher than that of younger elderly patients. This finding may be related to the relatively long course of disease in elderly patients, which leads to a more serious degree of steatosis. We also found that the CAP value in the younger population significantly increased following DAA treatment. The CAP value for elderly patients showed the same trend, but without statistical significance. There was no significant difference in CAP improvement between the two groups, which is consistent with the results of previous studies. We think this phenomenon could be explained, because elderly patients were always accompanied with sarcopenia and had worse nutritional status, but the specific mechanism between the two is still unclear. Therefore, hepatic steatosis improvement and changes in blood lipid levels after HCV clearance by DAAs should be more actively monitored [45]. Meanwhile, for the elderly, nutritional status assessment can be carried out during the treatment with DAAs, such as body composition analysis and skeletal muscle content determination and other relevant tests, to further clarify the potential causes of CAP changes in the elderly after DAAs treatment.

In this study, we also evaluated various factors associated with the regression of liver stiffness after the end of treatment in elderly patients. Among these factors, age, LSM at baseline, and CAP value were found to be independently associated with LSM improvement.

Almost all relevant studies have shown that LSM is an independent predictor for fibrosis regression in chronic HCV patients who achieve SVR after DAA treatment [41, 46, 47]. Similarly, our results indicate that the higher the baseline LSM, the better the improvement in fibrosis following DAA treatment. Considering that a higher baseline LSM value may reflect a higher degree of liver inflammation, improvements in liver enzyme indices for these patients should be more obvious, and the change in LSM should be more significant. In this study, we found that the CAP value was negatively and independently associated with fibrosis regression. Similarly, the work from Lackner C et al. also identified steatosis as a predictor for failed LSM improvement [48]. Soliman H et al. found that the steatosis degree, as measured by TE, was related to fibrosis regression in patients after receiving DAAs [49]. We determined that the hepatic steatosis of CHC patients may increase with DAA treatment, which may have a negative impact on hepatic fibrosis improvement and worsen long-term prognosis. At the same time, we emphasize that further research is needed to explore the mechanism that explains how degree of steatosis affecting fibrosis regression. Advanced age is also considered to be a predictor of poor prognosis. Therefore, on the premise of ensuring good health, it is important to begin antiviral treatment as early as possible. Recently, some studies have suggested that diabetes is a ‘bad trip companion’ of HCV, and that hyperglycemia is significantly associated with poorer stiffness regression [50, 51]. In the present work, we found that elderly patients were more likely to have diabetes than younger patients, so we included diabetes in our regression analysis. However, this term was not statistically significant.

In addition, factors such as low platelet counts, low or high ALT levels, high serum angiopoietin-2 levels, high FIB-4 score, and previous treatment history have also been identified as predictors associated with improved liver fibrosis [32, 47, 51–53]. However, these findings were not observed in this study. The impact of these predictors needs to be further evaluated in a larger population with a longer follow-up period.

This research has several limitations worth discussing. First, this study is retrospective in nature and has the potential for misinformation or missing data. To address these concerns, a more prospective study is necessary. Second, this study lacked histological evidence to examine the degree of liver fibrosis in CHC patients before and after DAA therapy. This primary reason for this was patient reluctance to undergo invasive procedures. Finally, the limited period of 12 weeks after treatment only allowed us to assess the temporary benefits of fibrosis after DAA therapy. Thus, future studies involving more patients and with longer follow-up periods are needed to better clarify the long-term benefits of DAAs in elderly patients.

In conclusion, LSM evaluated by TE, GPR, FIB-4, and APRI significantly decreased in elderly patients with CHC after HCV eradication. However, the degree of hepatic steatosis expressed by the CAP value did not significantly change as a result of treatment for the elderly group. Conversely, for younger patients, the CAP value significantly increased after DAA treatment. In addition, for elderly patients, we observed significant correlations between these three noninvasive serological evaluation markers and LSM, both before and after treatment. We found that age, LSM, and CAP at baseline were independent predictors of fibrosis regression in elderly CHC patients treated with DAAs. However, for elderly HCV patients at higher risk for liver fibrosis and HCC development, studies with more long-term follow-up periods are still necessary.

## Data Availability

All data generated or analysed during this study are included in this published article.

## Abbreviations

CHC: Chronic hepatitis C
HCV: Hepatitis C virus
PEG-IFN: Pegylated interferon
FDA: Food and drug administration
DAAs: Direct antiviral agents
LSM: Liver stiffness measurement
TE: Transient elastography
GPR: γ-Glutamyl transpeptidase to platelet ratio
APRI: Aspartate aminotransferase to platelet ratio index
FIB-4: Fibrosis-4
SVR: Sustained virological response
EOT: End of treatment
ALT: Alanine aminotransferase
AST: Aspartate aminotransferase
ALB: Albumin
γ-GT: Gamma-glutamyl transferase
TBil: Total bilirubin
WBC: White blood cells
PLT: Platelet
IQR: Interquartile range
HCC: Hepatocellular carcinoma
SOF: Sofosbuvir
RBV: Ribavirin
DCV: Daclatasvir
OBV/PTV/r/DSV: Ombitasvir/Paritaprevir/Ritonavir/Dasabuvir
ASV: Asunaprevir
DNVr: Danoprevir/Ritonavir
LEV/SOF: Ledipasvir/Sofosbuvir
EBR/GZR: Elbasvir/Grazoprevir
SOF/VEL: Sofosbuvir/Velpatasvir
HBV: Hepatitis B virus
HIV: Human immunodeficiency virus
GUCI: Göteborg university cirrhosis index
INR: Prothrombin time
PIIINP: Procollagen III N-terminal propeptide
MMP1: Matrix metalloproteinase-1
ARFI: Acoustic radiation force impulse
MRE: Magnetic resonance elastography
SREBP: Sterol regulatory element-binding protein
